# Removal notice to “Leveraging hybrid model of ConvNextBase and LightGBM for early ASD Detection via Eye-Gaze Analysis” [MethodsX 14 (2025) 103166]

**DOI:** 10.1016/j.mex.2026.103854

**Published:** 2026-03-03

**Authors:** Ranjeet Bidwe, Sashikala Mishra, Simi Bajaj, Ketan Kotecha

**Affiliations:** a Symbiosis Institute of Technology, Pune Campus, Symbiosis International (Deemed University), Lavale, Pune, Maharashtra, India; b School of Computer Data and Mathematical Sciences, University of Western Sydney (UWS), Sydney, Australia

This article has been removed: please see Elsevier Policy on Article Withdrawal (https://www.elsevier.com/about/policies-and-standards/article-withdrawal).

This article has been removed at the request of the Editor-in-Chief.

Post-publication, the journal was alerted to concerns regarding the dataset used in this study. It has been reported that the dataset contains images of patients without autism spectrum disorder (ASD) diagnosis. In addition, there is no information about informed patient consent.

The journal contacted the authors for clarification, and they stated that they were not aware of these concerns.

The Editor-in-Chief has determined that this article should be removed because the integrity and reliability of the study cannot be relied upon, and because some images from the dataset were republished without evidence of patient consent. Apologies are offered to the readers of the journal.

